# Infants Show Physiological Responses Specific to Parental Hugs

**DOI:** 10.1016/j.isci.2020.100996

**Published:** 2020-04-06

**Authors:** Sachine Yoshida, Yoshihiro Kawahara, Takuya Sasatani, Ken Kiyono, Yo Kobayashi, Hiromasa Funato

**Affiliations:** 1 Department of Anatomy, Faculty of Medicine, Toho University, Ota-ku, Tokyo 143-8540, Japan; 2 PRESTO, Japan Science and Technology Agency, Kawaguchi, Saitama 332-0012, Japan; 3 Graduate School of Engineering, The University of Tokyo, Bunkyo-Ku, Tokyo 113-8656, Japan; 4 Graduate School of Information Science and Technology, The University of Tokyo, Bunkyo-Ku, Tokyo 113-8656, Japan; 5 Graduate School of Engineering Science, Osaka University, Toyonaka, Osaka 560-8531, Japan; 6 International Institute for Integrative Sleep Medicine (WPI-IIIS), University of Tsukuba, Tsukuba, Ibaraki 305-8575, Japan

**Keywords:** Social Interaction, Psychosocial Factor, Human Physiology

## Abstract

Caregivers hug their infants to express affection and joy. However, it remains unknown how infants react to being hugged. Here we examined heart rate responses in first-year infants during a hug, hold, and tight hug from parents. Infants older than four months showed an increased R-R interval (RRI) during a hug, indicating reduced heart rates and pronounced parasympathetic activity. Few head movements predicted a higher RRI increase in infants during a parental hug compared with that during a hold and tight hug. Infants did not show an increased RRI during a hug from a female stranger. Infants younger than four months did not show RRI increase during parental hug but exhibited a decreased RRI correlated with contact pressure. Parents showed an increased RRI during hugging their infants. These results suggest the parent-infant hug underlies the parent-infant bonding and psychophysiological development of infants.

## Highlights

• We examined heart rate responses in first-year infants during hugs from parents
• Infants showed an RRI increase when hugged by parents but not by strangers
• Few head movements predicted higher RRI increase during a parental hug
• Parents also showed an increased RRI when hugging their infants

## Graphical Abstract

**Figure:**
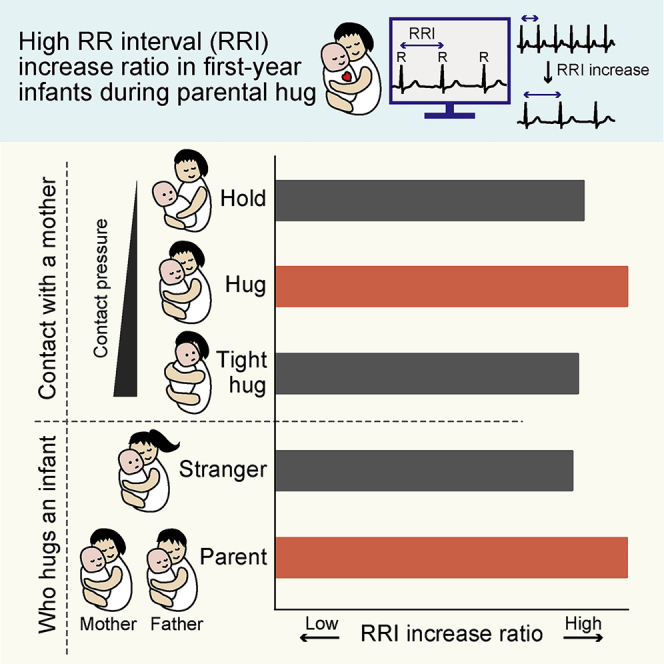

## Introduction

For a child, its relationship with its caregivers is crucial for both physical and mental development (Bowlby, 1969, Bowlby, 1977, Sullivan et al., 2011). Caregivers not only provide food and warmth to infants but also provide safety, proximity, and emotional bonding to them through physical interactions such as holding and hugging (Bowlby, 1969, Harlow, 1958). Both holding an infant and hugging an infant seem to be similar actions using parental arms, but the purposes of these behaviors are different. In general, although holding an infant has specific purposes such as moving and feeding, hugging an infant is an expression of caregiver's affection. In addition to the purpose, a hold and a hug differ in physical aspects such as postures and pressure. During the parent-infant hug, the contact area and pressure between the parent and infant are wider and stronger than those when the infant is held in the parent's arms for feeding or moving.

Because younger infants almost completely rely on other people, infants are frequently held by their parents. During breastfeeding, a mother needs to cradle her infant with her arms. Becasue similar nursing postures also work for formula feeding, being held with the hands is usually a routine position in which an infant is fed. Another purpose of a hold is to cause a calming response in infants. We showed that younger infants being held by their mothers while walking ceased crying and voluntary movement (Esposito et al., 2013). In contrast, a parent does not hug his/her infant for moving or feeding but to express positive feelings toward their infants, such as joy, love, happiness, and warmth. These feelings motivate caregivers to care for infants and lead to form emotional bonding with infants.

Given that the interaction between infants and their caregivers underlies the formation of mutual bonding and enhance emotional and intellectual development (Bowlby, 1977, Sullivan et al., 2011), the parent-infant hug may play a crucial role in forming an emotional relationship to significant others and a stable interpersonal relationship in a later stage of their life. Conversely, the developing emotional relationship between parents and infants may affect the physiological response to the parent-infant hug. However, to the best of our knowledge, there have been no reports about the physiological response to the parent-infant hug during the first year of life. Most studies examining parent-infant interactions were observational (Lotzin et al., 2015). It remains unknown how infants react to being hugged and being held or whether being hugged is comfortable for infants.

If a parent-infant hug works as social contact, the infant's response to parental hugs may be different from that to hugs from strangers. After 12 weeks, an infant is able to discriminate familiar and unfamiliar adults (Barrera and Maurer, 1981) and then, anecdotally, comes to enjoy being carried or hugged by a primary caregiver. Consistently, the parent-infant relationship may begin to show synchrony in gaze, vocal patterns, and heart rhythms as early as 3–5 months old (Feldman, 2007, Feldman et al., 2011).

To examine parent-infant social contact through body interactions, we focused on the physiological response during the parent-infant hug by measuring the pressure applied by the adult hand on the infant's back. To address whether the pressure between parent and infant during hugs can account for the physiological response in infants or whether a specific parent-infant relationship underlies the physiological response in infants, we conducted this study with infants' mothers, fathers, and female strangers.

Physiological parameters, such as heart rate and heart rate variability (HRV), have been used in many studies to examine sympathetic and parasympathetic activities (Chandola et al., 2010, Shaffer et al., 2017) in adults and children (Eyre et al., 2014, Herzig et al., 2017, Michels et al., 2013). HRV is a series of parameters assessing the variation in the time interval between successive R-R intervals (RRI) (Shaffer et al., 2017, Task Force of The European Society of Cardiology and the North American Society of Pacing and Electrophysiology, 1996). RRI is the reciprocal of the heartbeat rate, meaning that the RRI increase ratio (percentage of successive RRIs that are longer than the previous RRI) increases when the heartbeat rate decreases. HRV is usually measured in time or frequency domain parameters. Most HRV parameters require recordings of at least one minute to evaluate the autonomic regulation (Shaffer et al., 2017). However, it was almost impossible to avoid infant's bad mood during a 1-min or longer hold or hug. Moreover, HRV parameters do not evaluate trends in RRI changes during short-time actions. Thus, we used the RRI increase ratio to assess parasympathetic activity during a hold or hug that lasts for 20 s.

Here, we examined the RRI increase ratio in infants and their parents during parent-infant hug of the first-year infants. We observed an RRI increase, indicating parasympathetic activity, in infants during the mother-infant hug but not when infants were being held by a female stranger or during a very tight hug from their mother. A similar physiological change was also observed during the father-infant hug. Our findings will help us understand when and how social interaction between parents and prelinguistic infants develops during the first year after birth, as well as give a better understanding of the typical and atypical development of psychophysiological function.

## Results

### HRV during the First Year of Life

**Figure 1:**
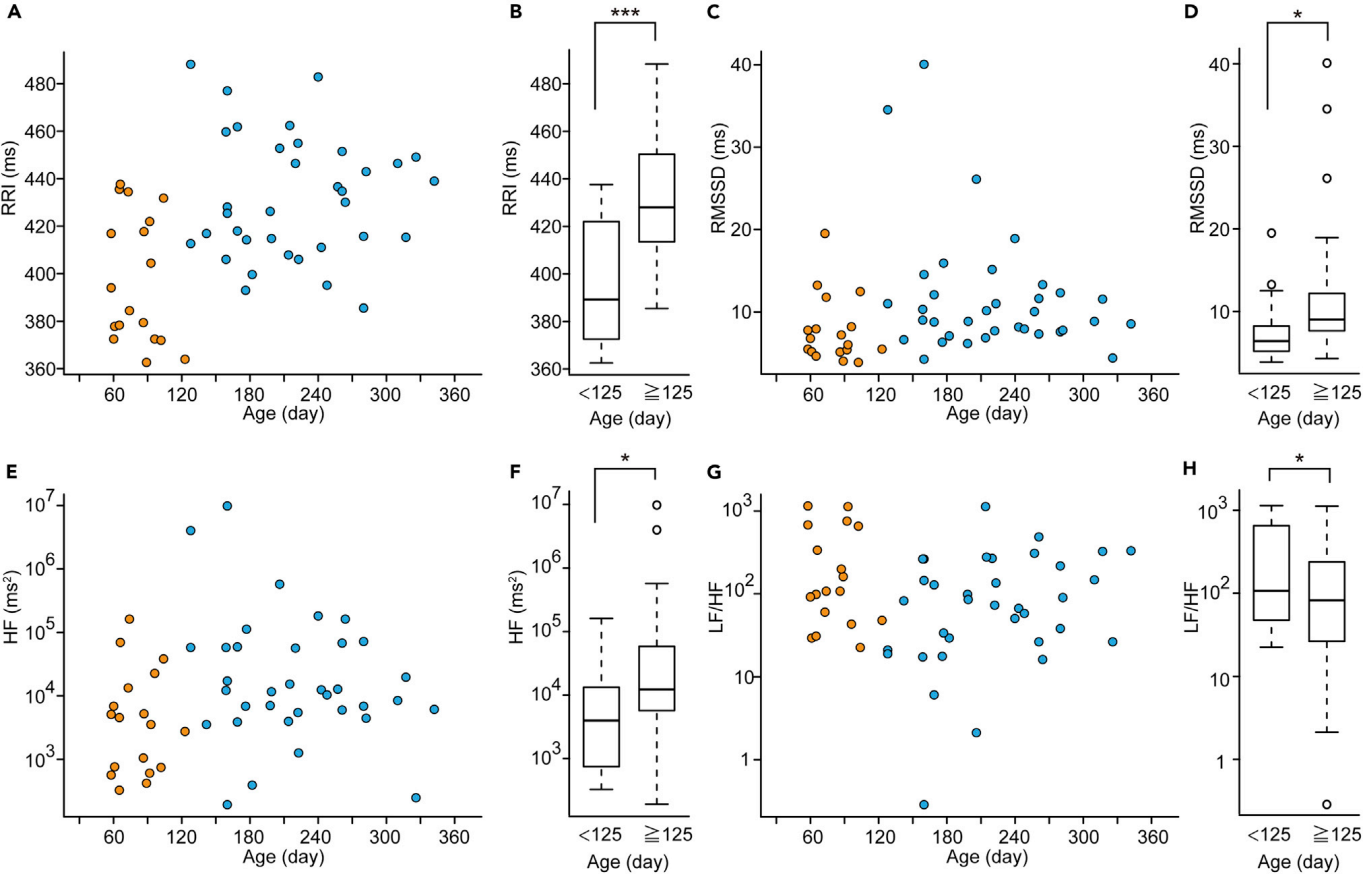
Heart Rate Variability in Undisturbed Infants in the Crib (A and B) The mean RRI of infants. (A) Individual values were shown (orange circles: younger than 125 days old, blue circles: older than 125 days old). (B) The RRI of infants older than 125 days old was higher than those younger than 125 days old. (C and D) The RMSSD of infants. (C) Individual RMSSD values were shown (orange circles: younger than 125 days old, blue circles: older than 125 days old). (D) The RMSSD of infants older than 125 days old was higher than those younger than 125 days old. (E and F) The HF of infants. (E) Individual HF values were shown (orange circles: younger than 125 days old, blue circles: older than 125 days old). (F) The HF of infants older than 125 days old was higher than those younger than 125 days old. (G and H) The LF/HF of infants. (G) Individual LF/HF values were shown (orange circles: younger than 125 days old, blue circles: older than 125 days old). (H) The LF/HF of infants older than 125 days old was lower than those younger than 125 days old. n = 53 (males = 28, females = 25). (B, D, F, and H) Welch's t test. The boxes represent the 25th, median, and 75th percentiles, and the whiskers represent the lowest or highest data within 1.5× interquartile range from the 25th or 75th percentile. ∗p< 0.05, ∗∗∗p< 0.001. See also Figures S1 and S2.

**Figure 2:**
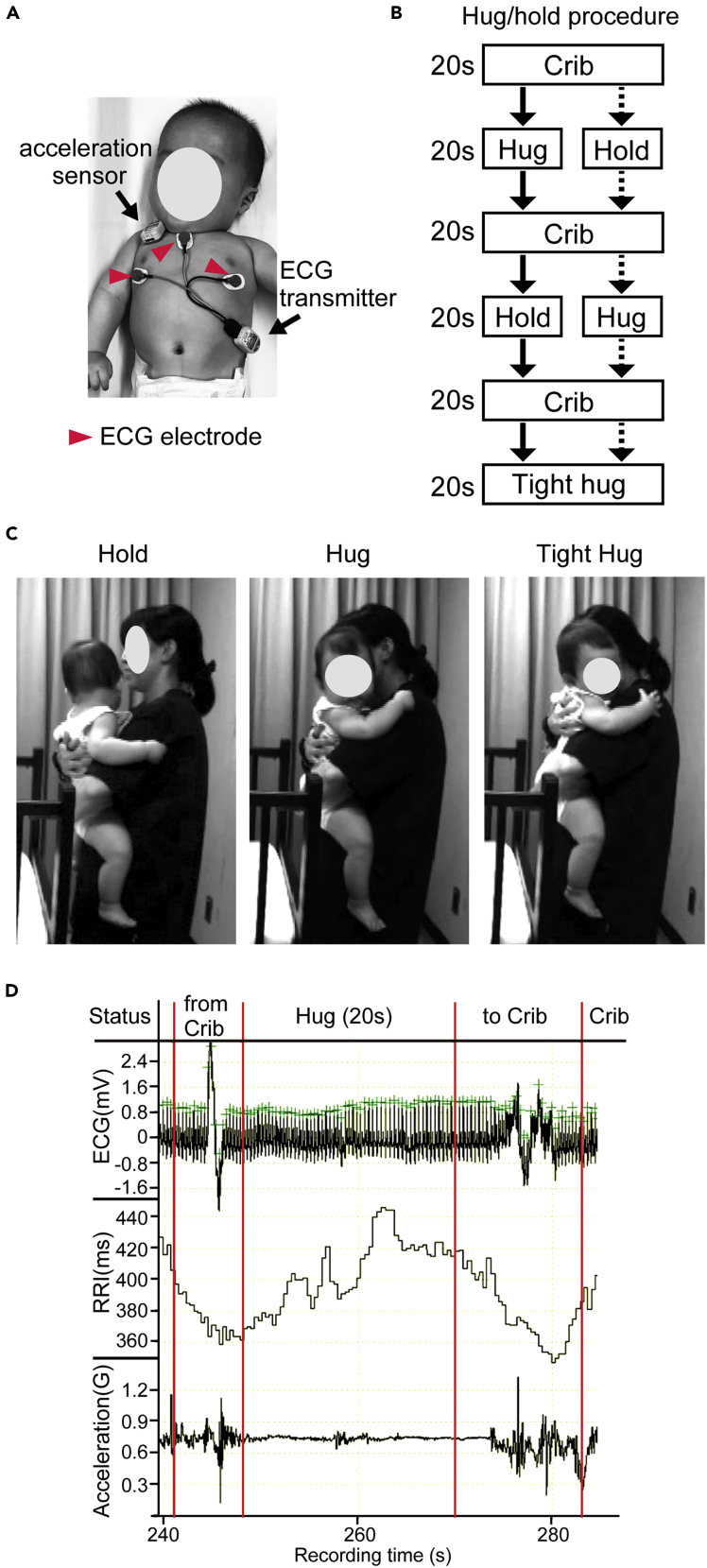
Hold/Hug Experimental Procedure (A) Three electrocardiogram (ECG) electrodes and an acceleration sensor were attached to the infant. (B) Procedure of the hug/hold experiment. Each task lasted for 20 s. Either of the two procedures (solid line arrow or dotted arrow) were randomly assigned. (C) Representative postures of mothers and infants during holding, hugging, and tight hugs. (D) Representative recording of ECG, R-R interval (RRI), and acceleration of an infant aged 317 days old while the mother lifted her infant from the crib, hugged her infant for 20 s, and then put her infant back down in the crib. The infant showed an increased RRI during the hug. The acceleration sensor detected the movement of the infant when being lifted from and lowered into the crib during the transition but did not detect movement during the hug. See also Figure S3.

First, we examined four major HRV parameters to clarify which HRV parameter was the most suitable to assess infants' autonomic system changes during a parental hug. The time domain parameters examined here were mean RRI and RMSSD (root-mean-square of successive differences between normal heartbeats). The frequency domain parameters were high-frequency power (HF) and the low-frequency power (LF)/HF ratio. Infants undisturbed in the crib showed a tendency to have an increased RRI or decreased heart rate during the first year of life (Figures 1A, 2A, and S1), which is consistent with previous reports (Harper et al., 1976, Katona et al., 1980). Because the RRI of very young infants was lower than that of older infants, we conducted classification and regression tree (CART) analysis to reveal that infants' RRIs could be divided into two groups at approximately 125 days old equivalent to the beginning of 4 months of age (Figures 1A and S2). The RRIs of infants older than 125 days old were larger than those of infants younger than 125 days old (Figure 1B, Welch's t test, t = 4.37, df = 33.22, p = 0.00011). In older infants, there was no significant correlation between days and the RRI (r = −0.030, p = 0.86), suggesting that the RRI remained stable between 4 and 12 months of age, at least in our dataset.

Infants tended to show increased RMSSD (Figure 1C) and HF (Figure 1E) and a decreased LF/HF (Figure 1G) during the first year, as previous reports on infants during quiet sleep have shown (Massin and von Bernuth, 1997, Patzak et al., 1996). Infants older than 125 days old showed an increase in the RMSSD (Figure 1D, Welch's t test, t = −2.45, df = 50.93, p = 0.018) and HF (Figure 1F, t = −2.57, df = 41.80, p = 0.014) and a decrease in the LF/HF (Figure 1H, t = 2.05, df = 41.63, p = 0.047) compared with those in infants younger than 125 days old. RMSSD and HF are mainly under the control of parasympathetic activity, and the LF/HF ratio has been proposed as a marker for autonomic nervous system balance (Task Force of The European Society of Cardiology and the North American Society of Pacing and Electrophysiology, 1996). These results indicate that parasympathetic activity increased in infants around four months old.

However, outliers could not be avoided in these parameters, and frequency domain parameters were generally not suitable for the short duration tasks of 20 s as in this study. Thus, among RRI, RMSSD, HF, and LF/HF, we used the RRI increase parameter to assess a physiological response in infants thereafter. We adopt an RRI increase ratio rather than the mean RRI, as a representative value to clarify the dynamics of whether heart rates tended to increase or decrease during a hug. We analyzed infant RRI increase ratio data separately according to whether the infants were older or younger than 125 days old in this study.

### RRI Increases in Infants during Parental Hug

To examine physiological responses during mother-infant hugs, mothers were instructed to randomly hug or hold their infants with ECG electrodes and an acceleration sensor (Figures 2A and 2B) and then give them a very tight hug (Figures 2C and S3). Importantly, the mother was instructed not to hug infants mechanically but to hug them with positive affection. RRI was monitored during this hug/hold procedure (Figure 2D), and the RRI increase ratios during a hold, hug, and tight hug were evaluated.

In infants younger than 125 days old, multiple regression analysis of the RRI increase ratio yielded significant regression coefficients for the hug/hold task (r^2^ = 0.14, F [5, 74] = 2.50, p = 0.038, β = −0.30, p = 0.0057) but not for sex, body weight, being the first child, or previous task type (Table S1). There was no significant difference in the RRI increase ratio, depending on whether the hug/hold was performed first (Hold, Welch's t test, t = 1.053, df = 22.73, p = 0.30; Hug, Welch's t test, t = 1.059, df = 10.75, p = 0.31). Similarly, in infants older than 125 days old, multiple regression analysis yielded significant regression coefficients for the hug/hold task (r^2^ = 0.067, F [5, 213] = 3.061, p = 0.011, β = −0.21, p = 0.0023) but not for sex, body weight, being the first child, or previous task type (Table S1). There was no significant difference in the RRI increase ratio, depending on whether the hug/hold was performed first (Hold, Welch's t test, t = 1.13, df = 41.15, p = 0.26; Hug, Welch's t test, t = −0.16, df = 48.48, p = 0.87). Preliminary confirmation revealed that there were no significant difference in the respiration rate in the present hug/hold tasks (Welch's t test, t = 0.079, df = 17.99, p = 0.94 in infants younger than 125 days, t = −0.53, df = 44.14, p = 0.60 in infants older than 125 days).

**Figure 3:**
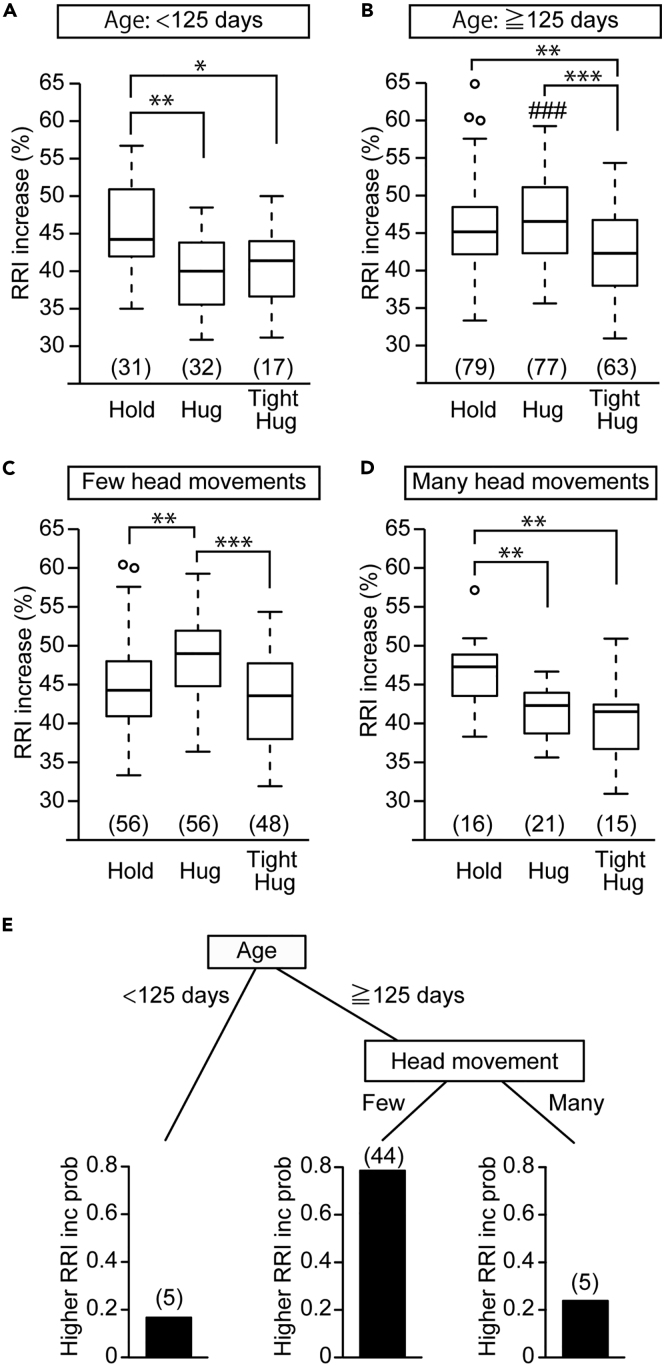
Prediction of Infant's Heart Rate Changes during Mother-Infant Hug by Head Movements (A and B) The RRI increase ratio of infants younger (A) or older (B) than 125 days old during holding, hugging, or tight hugs from his/her mother. (C and D) The RRI increase ratio of infants older than 125 days old during a hug from his/her mother was compared depending on whether the infant showed few head movements (C) or many head movements (D) in the crib immediately before hugging. (A–D) Welch's ANOVA followed by pairwise comparisons using Welch's t test with Holm's correction. (E) CART analysis for the RRI change using infants' age and the head movement as independent variables. prob: probability. The boxes represent the 25th, median, and 75th percentiles, and the whiskers represent the lowest or highest data within 1.5× interquartile range from the 25th or 75th percentile. Numbers in parentheses indicate the number of infants.∗p< 0.05, ∗∗p< 0.01, ∗∗∗p< 0.001. ^###^p<0.001, vs. <125 days. See also Figure S4, Tables S1 and S2.

Infants younger than 125 days old showed a lower RRI increase ratio during both a hug and a tight hug than during a hold (Figure 3A, Welch's ANOVA, F[2, 42.68] = 8.82, p = 0.00062, pairwise comparisons, p = 0.00033 in hold versus hug, p = 0.029 in hold versus tight hug, p = 0.38 in hug versus tight hug). In contrast, the RRI increase ratios of infants older than 125 days old during a hug and a hold were similar (Figure 3B, Welch's ANOVA, F[2, 141.30] = 9.92, p = 0.000093, pairwise comparisons, p = 0.53 in hold versus hug) and were higher than that during a tight hug (pairwise comparisons, p = 0.0013 in hold vs. tight hug, p = 0.00014 in hug versus tight hug). The RRI increase ratio in infants older than 125 days old was higher than that in infants younger than 125 days old during a hug but not during a hold or a tight hug (Figure 3A and 3B, Welch's t test, t = −6.43, df = 41.64, p < 0.0001 in hug, t = 0.91, df = 33.87, p = 0.37 in hold, t = −1.34, df = 13.16, p = 0.20 in tight hug). In other words, the altered RRI response in infants was dependent upon whether they were younger or older than 125 days old and was only recognized during a hug.

During this study, we noticed that infants showed large individual differences in RRI responses to hugs and that infants who did not move their head immediately before being hugged tended to show an increased RRI during the mother-infant hug. To examine this relationship, we divided infants into either a few head movements or many head movements groups. Older infants making few head movements showed a higher RRI increase ratio during a hug from their mother than during a hold (Figure 3C, Welch's ANOVA, F[2, 102.39] = 12.23, p < 0.0001, pairwise comparisons, p = 0.0039 in hold versus hug, p = 0.13 in hold versus tight hug, p < 0.0001 in hug versus tight hug). In contrast, infants making many head movements showed a lower RRI increase ratio during a hug than during a hold (Figure 3D, Welch's ANOVA, F[2, 28.032] = 9.12, p = 0.00089, pairwise comparisons, p = 0.0018 in hold versus hug, p = 0.0023 in hold versus tight hug, p = 0.41 in hug versus tight hug). None of the upper body movements, lower body movements, or vocalizations were associated with RRI differences between holds and hugs (Figure S4). Independent CART analysis also found that the RRI increase ratio was predicted if both infants' ages and head movement types were provided (Figure 3E, overall accuracy 83.18%), when RRI increase ratio was binarized depending on whether it was higher or lower than the reference value (see Transparent Methods). After 125 days, the probability of a higher RRI increase was 0.79 in infants making few head movements (Figure 3E). In contrast, the probability of a higher RRI increase was 0.24 in infants making many head movements (Figure 3E). This result also suggests that the RRI increase ratio during a hug was lower in infants younger than 125 days old regardless of the head movement type (Figure 3E). The probability was 0.17 in infants younger than 125 days old. In older infants, multiple regression analysis yielded significant regression coefficients for the head movement type (Table S2, r^2^ = 0.28, F [5, 71] = 5.54, p = 0.00023, β = −0.51, p < 0.0001) but not for sex, body weight, order of hugs during the hold/hug task, or being the first child (Table S2).

### Few Head Movements Predict RRI Increase during a Parental Hug

Next, we examined whether the head movements of older infants can be a predictor of RRI response, not only during maternal hugs but also during paternal hugs. The ratio of older infants who showed few head movements before a hug was similar for hugs from mothers and fathers (72.7% before a maternal hug, 69.2% before a paternal hug, Fisher's exact probability test, p = 0.75). This result suggests that whether a mother or father was going to hug the infant does not affect infant head movement frequency.

**Figure 4:**
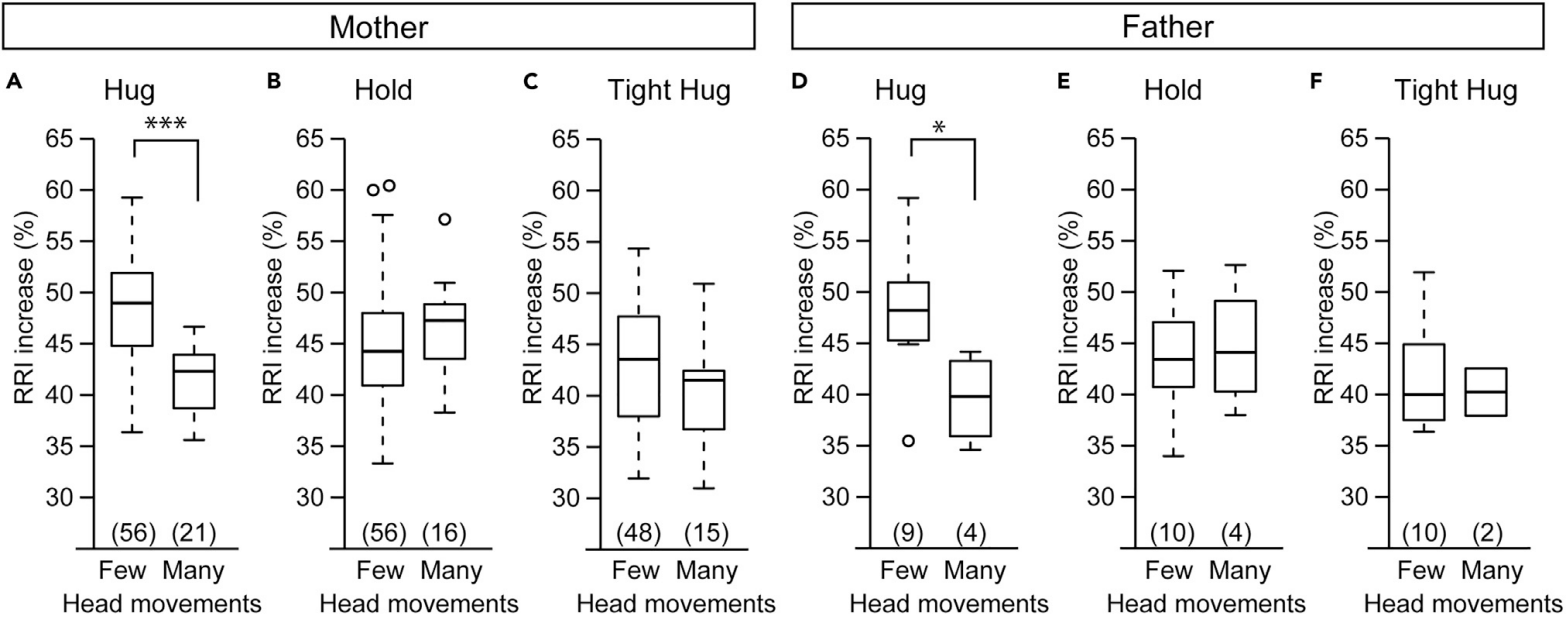
Infant's Heart Rate Changes during Parent-Infant Hug (A–C) The RRI increase ratios of infants older than 125 days old during a hug (A), a hold (B), and a tight hug (C) from his/her mother were compared depending on whether the infant showed few or many head movements in the crib immediately before each task. (D–F) The RRI increase ratios of infants older than 125 days old during a hug (D), a hold (E), and a tight hug (F) form his/her father were compared depending on whether the infant showed few or many head movements in the crib immediately before each task. (A–F) Welch's t test. The boxes represent the 25th, median, and 75th percentiles, and the whiskers represent the lowest or highest data within 1.5× interquartile range from the 25th or 75th percentile. Numbers in parentheses indicate the number of infants. ∗p< 0.05, ∗∗∗p< 0.001.

In maternal hugs, older infants with few head movements showed a higher RRI during the hug than that in those with many head movements (Figure 4A, t = 6.37, df = 56.70, p < 0.0001). There was no significant difference in the RRI increase ratio during a hold or a tight hug (Figure 4B, t = −1.46, df = 34.19, p = 0.15, Figure 4C, t = 1.62, df = 24.76, p = 0.12). Similarly, in paternal hugs, the RRI increase ratio was higher in infants with few head movements than in those with many head movements (Figure 4D, t = 2.73, df = 8.71, p = 0.024). There was no significant difference in the RRI increase ratio during a hold or a tight hug (Figure 4E, t = −0.26, df = 4.84, p = 0.80, Figure 4F, t = 0.60, df = 2.21, p = 0.60). The RRI increase ratio during the crib immediately before hugs showed no significant difference between few and many head movement groups (t = −0.63, df = 54.13, p = 0.53 in maternal hugs, t = −0.039, df = 6.073, p = 0.97 in paternal hugs). Importantly, there was also no significant difference in the RRI increase ratio between maternal and paternal hugs for either head movement type (t = 0.064, df = 9.72, p = 0.95 in few head movement, t = 0.94, df = 3.69, p = 0.41 in many head movement). Thus, the head movements of older infants predict the RRI response during a parent-infant hug.

### Infants Showed Different RRI Responses to a Stranger

**Figure 5:**
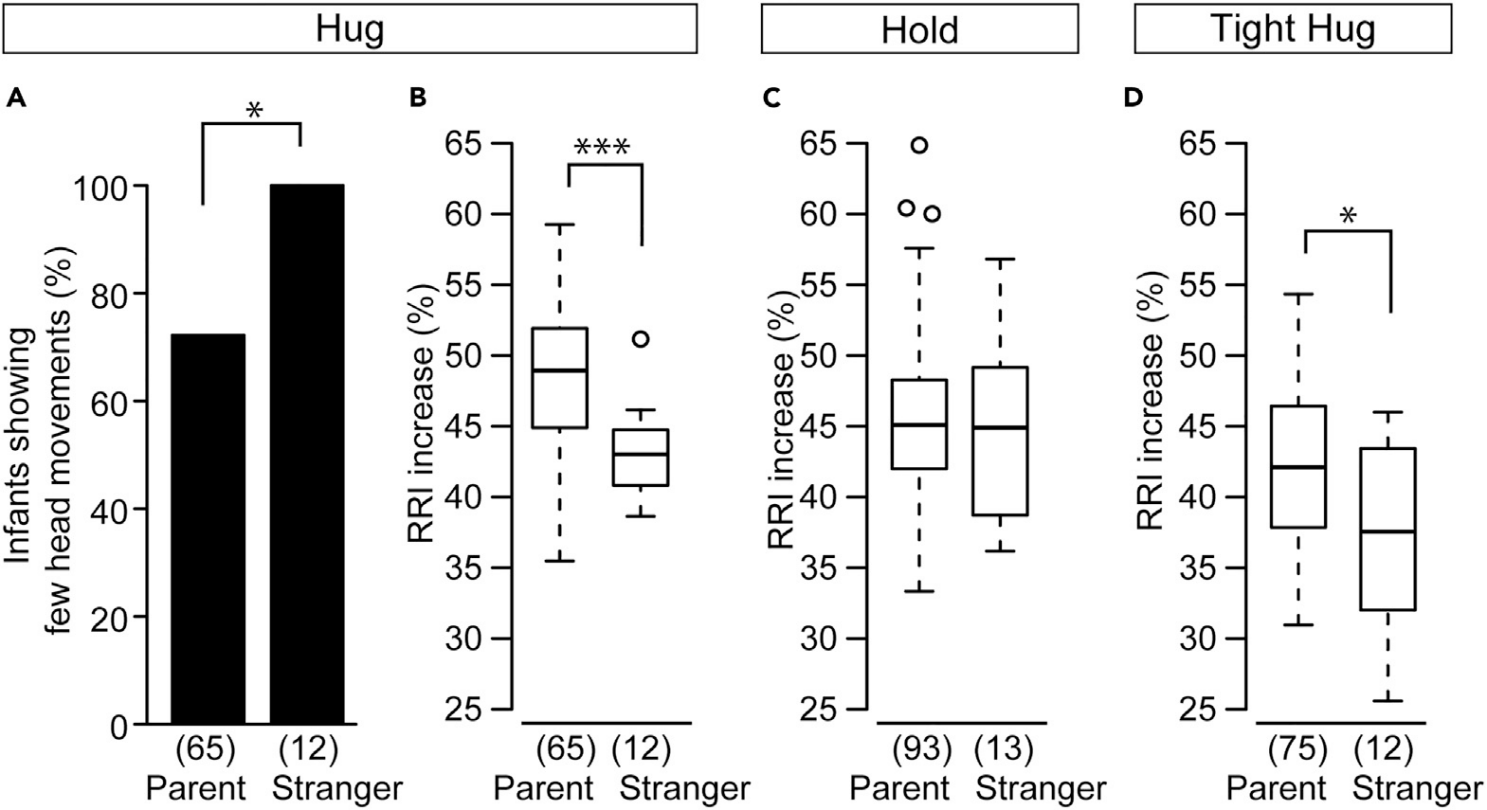
Infant's Heart Rate Changes during a Hug from a Female Stranger (A) The ratio of older infants that showed few head movements in the crib immediately before hugging was compared between the parents and female strangers. Fisher's exact probability test. (B–D) The comparison of the RRI increase ratio in infants more than 125 days old during a hug (B), a hold (C), and a tight hug (D) between experiments with parents and female strangers. Welch's t test.The boxes represent the 25th, median, and 75th percentiles, and the whiskers represent the lowest or highest data within 1.5× interquartile range from the 25th or 75th percentile. Numbers in parentheses indicate the number of infants. ∗p< 0.05, ∗∗∗p< 0.001.

Next, we examined whether the infant's high RRI increase ratio during a parent-infant hug was also observed when the older infants were hugged by a female stranger who the infant had not met previously and who had experience in childbirth and parenting. During the hold/hug session with female strangers, none of the infants showed many head movements before the hug, because most infants kept looking at the stranger or the area where the infant had seen their mother the last time (Figure 5A, Fisher's exact probability test, p = 0.035). This result suggests that the head movement of the infants changed depending on their social cognition and environment. Notably, the RRI increase ratio was lower during the hug from a female stranger than during hugs from parents despite there being few head movements (Figure 5B, Welch's t test, t = 4.33, df = 23.48, p = 0.00024), indicating that few head movements are not a good predictor of a high RRI increase during a hug from a female stranger. The RRI increase ratio during a hold was similar between female strangers and parents (Figure 5C, Welch's t test, t = 0.45, df = 14.50, p = 0.66). In contrast, the RRI increase ratio during a tight hug from female strangers was lower than that during tight hugs from parents (Figure 5D, Welch's t test, t = 2.50, df = 13.35, p = 0.026). Thus, the RRI changes in infants during a hug and a tight hug were different depending on whether they were received from parents or strangers.

### Relationship between Infants' Heart Rate and Contact Pressure

**Figure 6:**
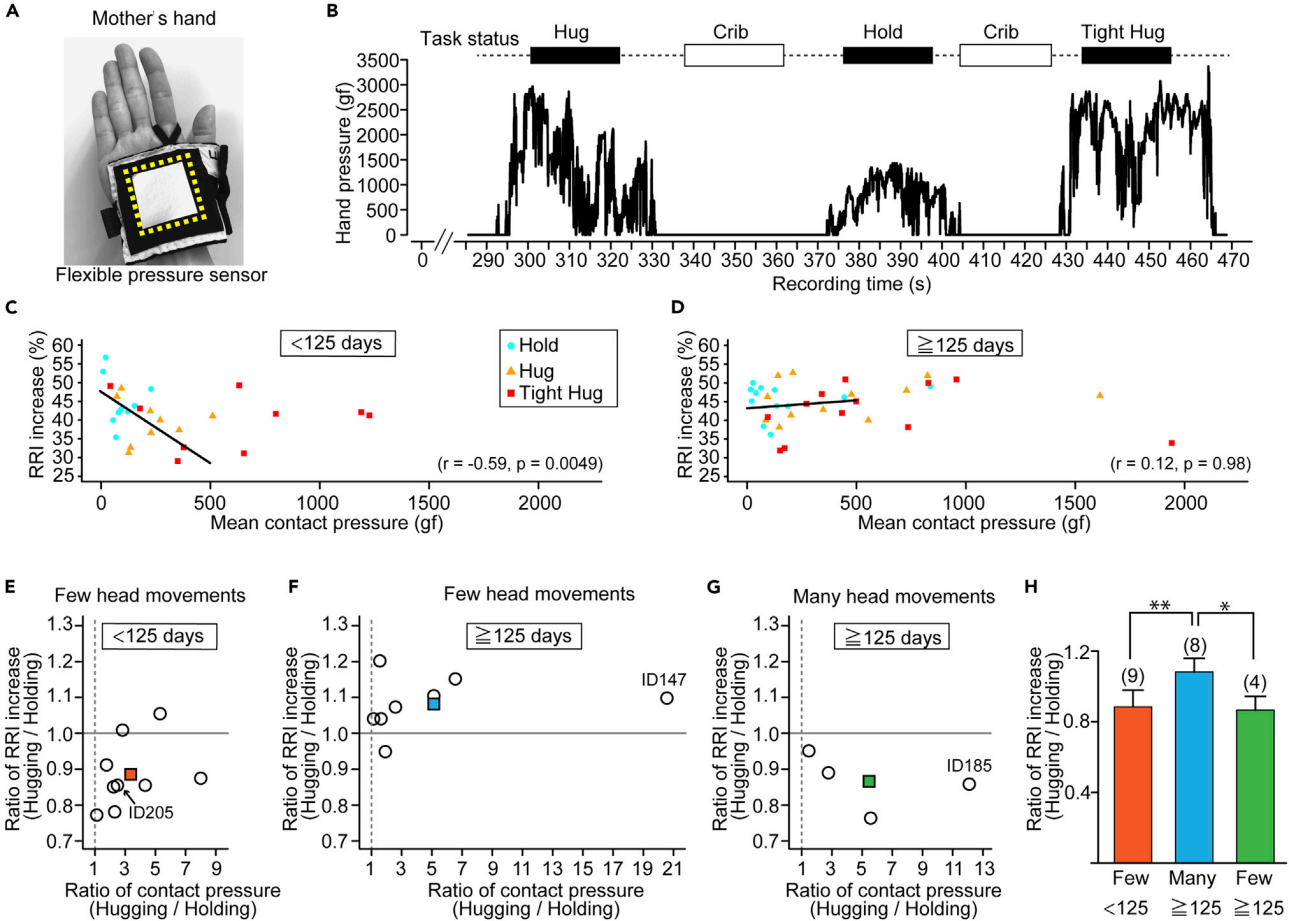
Relationship between the Contact Pressure from Mother's Hand and the Heart Rate of the Infant (A) A pressure sensor was attached to the palm of the mother's hand. The yellow dotted square indicates the pressure sensor. (B) Representative pressure changes during a single hug/hold session. The pressure was lowest during the holding and largest during the tight hug. (C and D) Relationship between the mean contact pressure during a hold, a hug, and a tight hug and the RRI increase ratio in infants younger than 125 days old (C) (n = 9) and older than 125 days old (D) (n = 12). The black line indicates a regression line calculated from the RRI increase ratio and a mean contact pressure less than 500 gf. Linear regression analysis. (E–G) The ratio changes in the RRI increase ratio and the mean contact pressure from a hold to a hug in the same infants younger than 125 days old with few head movements (E) and in those older than 125 days old with few head movements (F) and with many head movements (G). Each circle represents each infant data. Circles with ID number indicate infants who are seen in Supplementary Videos (ID205 (E) in Video S1, ID147 (F) and ID185 in Video S2). A colored square in each graph indicates the mean (orange square (E), blue square (F), and green square (G)). (H) The comparison of the ratio changes in the RRI increase ratio in infants younger than 125 days with few head movements (orange) and in those older than 125 days with few head movements (blue) and with many head movements (green). MANOVA followed by one-way ANOVA and the Tukey-Kramer post-hoc test. Numbers in parentheses indicate the number of infants. Mean ± SD. ∗p< 0.05, ∗∗p< 0.01. See also Figures S5 and S6.

We examined how physical pressure between the mother and infant affects RRI. We measured the contact pressure between the infant's back and the mother's hand during the hold/hug session using a flexible pressure sensor attached to the mother's hand (Figure 6A). In most cases, each mother-infant pair showed increasing mean pressure for hold, hug, and tight hug, successively (Figure 6B). The mean contact pressures during a hug and a tight hug shifted toward the higher side compared with during a hold in both younger infants (Figure S5A, Wilcoxon rank-sum test, p = 0.038 in hold versus hug, p = 0.0083 in hold versus tight hug) and older infants (Figure S5B, Wilcoxon rank-sum test, p = 0.020 in hold versus hug, p = 0.0087 in hold versus tight hug). Notably, there was no significant correlation between the body weight of infants and the mean contact pressure during a hold, hug, and tight hug (Figure S6).

For infants younger than 125 days old, we found a negative correlation between the mean contact pressure and the RRI increase ratio when the contact pressure was lower than 500 gf (Figure 6C, r = −0.59, p = 0.0049). None of the younger infants showed many head movements. In contrast, infants older than 125 days old did not show any correlation between the mean contact pressure and the RRI increase ratio (Figure 6D, r = 0.12, p = 0.98). For each mother-infant pair, the ratio of RRI increase during a hug to during a hold and the ratio of mean contact pressure during a hug to during a hold were plotted (Figures 6E–6G). This plot clearly showed that younger infants with few head movements showed a lower RRI increase ratio (mean ± SEM; 0.88 ± 0.032, Figure 6E, Video S1 showing the infant identification number(ID) 205), whereas older infants with few head movements showed the opposite result (mean ± SEM; 1.082 ± 0.027, Figure 6F, the first half of Video S2 showing the infant ID 147). Older infants with many head movements showed a lower RRI increase ratio (mean ± SEM; 0.87 ± 0.039, Figure 6G, the latter half of Video S2 showing the infant ID 185). Older infants with few head movements hardly moved during a hug, whereas older infants with many head movements often started moving vigorously during a hug (the representative movement gradually became clear around 21 s in Video S2). Multivariate analysis of variance (MANOVA) followed by one-way ANOVA and the Tukey-Kramer post-hoc test supported the finding that older infants with few head movements showed a higher RRI increase ratio during a hug than older infants with many head movements and younger infant groups (Figure 6H, MANOVA, Wilks-Lambda = 0.38, F[4, 34] = 5.34, p = 0.0019; Tukey-Kramer post-hoc tests, p = 0.017 in older infants with few head movements versus older infants with many head movements; p = 0.0044 in older infants with few head movements versus younger infant with few head movements; p = 0.93 in younger infants with few head movements versus older infants with many head movements). Thus, many head movements are a good predictor for RRI increase during a hug in infants older than 125 days old.

Video S1. Changes in RRI and Contact Pressure in an Infant Younger than 125 days Old Who Showed Few Head Movements before Hold and Hug, Related to Figure 6 This video shows behavioral, RRI, and contact pressure changes in the same male infant aged 80 days old (Infant identification number (ID) 205 in Figure 6E) from the end of the transition period to the first 17 s of the hug or hold period. The upper and lower panels show a set of infant RRI (yellow) and contact pressure (blue) changes during a hug and hold, respectively. Pale color shading means a hug (upper) or hold (lower) period. At the end of the video, RRI increase ratio (%) and mean contact pressure (gf) of the task are presented in the right-upper corner of the RRI trend and pressure graphs, respectively. bpm: beats per minute.

Video S2. Changes in RRI and Contact Pressure during a Hug in an Infant Older than 125 days Old, Related to Figure 6 This video shows behavioral, RRI, and contact pressure changes in two different infants (Infant identification number (ID) 147 in Figure 6Fand ID185 in Figure 6G) from the end of the transition period to the first 12 s of the hug period. Infant number 147 and 185 are 206-day-old male and 285-day-old female infants, respectively. The upper and lower panels show a set of infant RRI (yellow) and contact pressure (blue) changes in infants who showed few head movements (upper) and many head movements (lower), respectively. Pale color shading means a hug period. RRI increase ratio (%) and mean contact pressure (gf) of the task are presented in the right-upper corner of the RRI trend and pressure graphs, respectively. bpm: beats per minute.

### Parent RRI Changes when Hugging One's Own Infant

**Figure 7:**
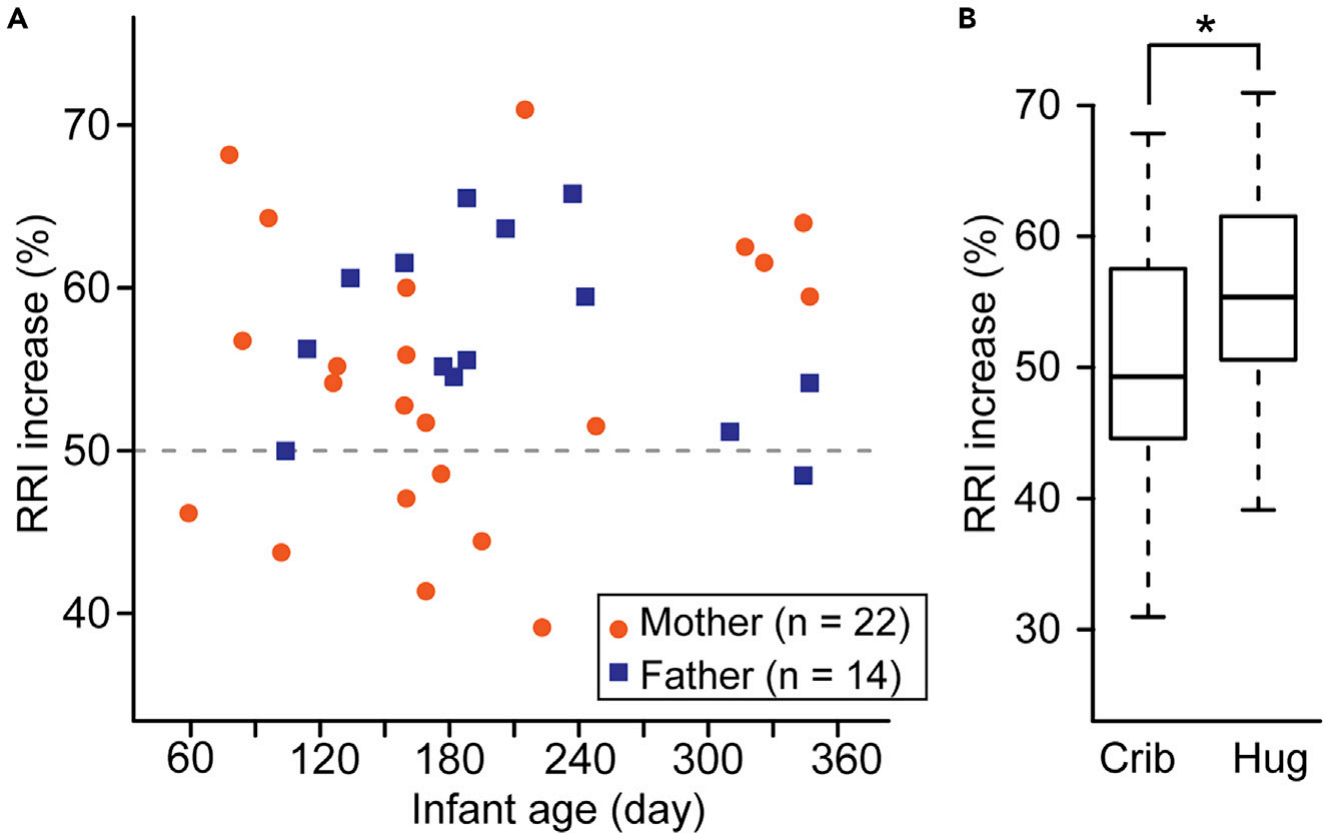
Parent's Heart Rate Changes in Response to Hugging His/Her Infant (A) The RRI increase ratio of the mothers (orange circles) and fathers (dark blue rectangles) when hugging his/her infant. (B) The RRI increase ratio of parents (n = 36) during the hug was higher than that in the crib immediately before hugging. Welch's t test. The boxes represent the 25th, median, and 75th percentiles, and the whiskers represent the lowest or highest data within 1.5× interquartile range from the 25th or 75th percentile. ∗p< 0.05. See also Table S3.

Because parent-infant hug is an interactive behavior, we examined whether the hug affects the psychological and physiological aspects of parents. Using a questionnaire, we asked mothers and fathers whether they felt relieved when hugging their infants. Our investigation revealed that more than 90% of the mothers and fathers reported feeling relieved when they hugged their infants (100% in mothers (n = 25), 91.30% in fathers (n = 23)). Finally, we examined how the parent-infant hug affected parent RRI. Most of the mothers and fathers exceeded a 50% RRI increase ratio during the hug regardless of the age of the infant (Figure 7A, 68.18% in mothers, 93.86% in fathers). The RRI increase ratio of neither mothers nor fathers showed a significant correlation with infant age (Figure 7A, r = 0.23, p = 0.30 in mothers, r = −0.27, p = 0.35 in fathers). The RRI increase ratio of the parents during a hug was higher than that before the hug (Figure 7B, Welch's t test, t = −2.49, df = 35, p = 0.018). There was no significant difference between maternal and paternal RRI increase ratios during the hug (Welch's t test, t = −1.15, df = 33.99, p = 0.29) and immediately before the hug (Welch's t test, t = 0.18, df = 30.33, p = 0.86). Thus, the parent-infant hug may relax the autonomic system and affect the psychological responses of the parents.

## Discussion

In this study, we showed that (1) infants older than 125 days old exhibited different RRI reactions during a hug depending on whether parents or stranger hugged them, (2) few head movements before a parental hug were associated with RRI increases in infants older than 125 days old, (3) infants younger than 125 days old exhibited RRI changes correlated with the amount of pressure placed on the body, and (4) a parent-infant hug increased the RRI of the parents. A hug behavior may start functioning as an emotional bonding between parents and prelinguistic infants at four months after birth. To the best of our knowledge, this study is the first quantitative and cross-sectional report investigating hugging behavior between parents and infants during their first year.

Infants older than four months old showed a higher RRI increase ratio when they were hugged by their parents than by female strangers. Although the recognition of infants that they are being hugged by their parents is required for the RRI increase, just being held by their parents or receiving a very tight hug from their parents did not lead to an RRI increase. This finding indicates that the physical contact with their parents is not enough to trigger a heart rate response and that being hugged by their parents has a special relaxing effect on infants through the way they are hugged, as well as through who is hugging them. The pressure level during the parent-infant hug that makes infants feel relieved and comfortable varies across parent-infant relationships. We think that different infants' responses toward their parents and strangers may reflect infants' feelings of relief when they are hugged by their parents and/or infants' feelings of tenseness when they are hugged by a female stranger. Bonobos (*Pan paniscus*), our closest living primate relatives, also hug a companion to console (Clay and de Waal, 2013). Interestingly, mother-reared juvenile bonobos are more likely to show consolation behaviors than orphaned juveniles, suggesting the critical role of early experience between the caregiver and infant in the development of socioemotional interaction. RRI changes in infants being hugged may be one of the fundamental physiological responses that foster the sense of acceptance and empathy in primates.

The comfortable pressure during the parent-infant hug may not be simply determined by the bodyweight of the infant or the muscle strength of the parents' arms. We observed higher contact pressure from the mother's hand on her infant's back for holds, hugs, and tight hugs, in that order. Cutaneous receptors mature at approximately 4–7 weeks of gestation, and somatosensory function follows thereafter (Bremner and Spence, 2017); thus both younger and older infants may be able to discriminate against the pressure differences between a hold, a hug, and a tight hug.

The heart rate and HRV data of the infants in this study are consistent with delayed parasympathetic activity maturation (Chatow et al., 1995, Eyre et al., 2014, Massin and von Bernuth, 1997, Silvetti et al., 2001). Furthermore, we showed that the maturation of parasympathetic activity progresses rapidly by the end of 4 months and remains almost stable between 4 months and 12 months of age. Thus, increased parasympathetic activity may lead to a higher RRI increase ratio during the maternal hug in infants aged four months old and older. In contrast, sympathetic activity dominance in infants younger than four months old accounts for the decreased RRI correlated with increased contact pressure placed upon infants. The pressure placed upon the infants during hugs resulted in enhanced sympathetic activity and a lower RRI increase ratio. The finding that a tight hug from parents reduced RRIs in both younger and old infants suggests that pressure that is too tight leads to enhanced sympathetic activation. Because the preliminary experiments showed that the infants tended to become fussy during a tight hug, we conducted a tight hug last. However, it is possible that infants were getting tired at the last task of the procedure, which affects RRI changes during a tight hug.

It is unlikely that the RRI decrease during a mother-infant hug in infants younger than 4 months old is due to infants' inability to recognize their mother because 3-month-old infants are able to discriminate between their mothers' faces and faces of strangers (Barrera and Maurer, 1981, deHaan et al., 2003, Halit et al., 2004). From the age of three months old, infants are able to interact with their mother through gaze, facial expression, and pre-conversation-like vocal activity (Feldman et al., 2011). Some studies have shown that newborns already prefer the face of their mother over that of a stranger (Bushneil et al., 1989, Field et al., 1984, Pascalis et al., 1995).

What we showed in the previous study (Esposito et al., 2013) was a strong calming effect of being carried by a walking mother, which made infants stop crying immediately. We suppose that sensory input from the vestibular system responding to acceleration and position of head and body may lead to the calming effect of being rocked. In contrast, we designed our hug/hold session to exclude the effect of the vestibular system. As predicted, the relaxing effect of being hugged by a parent who stood still that we examined here was much milder than being carried and could not make infants stop crying (S.Y., unpublished data).

In adults, discriminative and emotional aspects of tactile stimuli are processed in different parts of the brain (McGlone et al., 2014, Olausson et al., 2002). The orbitofrontal cortex (Francis et al., 1999), anterior cingulate cortex (Case et al., 2016), and superior temporal sulcus (Davidovic et al., 2016) are involved in the process of pleasantness of touch. However, little is known about the cortical processing of the emotional features of tactile stimuli in infants and this needs to be examined in future research.

Regarding sensory modalities other than pressure and posture, we designed our holding/hugging task to reduce complications due to smell, sound, vibration, texture, temperature, and touch sensation, trying to make these differences as small and stable as possible. Because these sensory inputs were almost stable during hold, hug, and tight hug, the increased RRI specifically associated with the parental hug is thought to be mainly caused by the pressure and posture on which infants rely (McGlone et al., 2014). However, we do not mean that olfactory and visual information from parents have nothing to do with infant's RRI response but suggest that other sensory modalities work together with pressure sense to trigger infant's RRI response. In addition, we cannot deny the possibility that unfamiliar visual and/or olfactory inputs suppressed infant's RRI changes during being hugged by female strangers.

We also found that few head movements predicted a high RRI increase ratio in older infants but not in younger infants. In other words, frequent head movements in older infants may predict shorter RRIs or increased sympathetic activation. We thought that infants older than four months old moved their heads for some purpose or due to curiosity regarding their surroundings and were, thus, unhappy and agitated when they were interrupted by the maternal hug (Video S2). In fact, infants become more active in terms of voluntary movement and less active in terms of certain spontaneous movements and general movement at approximately 120 days old (vonHofsten, 1984, Kanemaru et al., 2012, Prechtl, 1997, Prechtl and Hopkins, 1986). Interestingly, no infants moved their head frequently when hugged by a female stranger. Instead, the infants kept attentively looking at the stranger, which supports the idea that the head movements of older infants represent infants' visual attention toward their surroundings. Also, the presence of parents or a stranger may affect the emotions and exploratory behaviors of infants. Although infants actively showed exploratory behavior when their mothers were present as a “secure base,” the presence of a stranger quickly reduced such exploratory behavior (Ainsworth and Bell, 1970). Thus, an infant's level of head movement may work well as a handy and useful index for social cognition, as well as motor development, in the interaction between parents and infants in daily life.

Oxytocin, which is sometimes referred to as the “hug hormone,” because frequent partner hugs have been associated with high oxytocin levels (Light et al., 2005), plays an important role in the parent-infant relationship (Feldman, 2017, Feldman et al., 2010, Gordon et al., 2010). Although it is unlikely that oxytocin is involved in the heart rate responses during the parent-infant hug, because the quick responses that occur in less than 20 s are hardly triggered by hormonal regulation, we suppose that oxytocin shapes the parent-infant relationship that underlies the parent-specific parasympathetic activation in infants.

The parents showed significantly higher RRI increase ratio during hugging than before hugging regardless of the age of their infants, suggesting that hugging their infant makes parents calm. We think the current study showed that the parent-infant hug is an interactive behavior that promotes forming mutual bonding between parents and prelinguistic infants and exhibits a calming effect on parents and older infants. The relaxing effect often allows parents to hug their infants spontaneously rather than obligatorily. Infants may get used to being hugged and may be able to react to hugging depending on their emotional state and the specific interpersonal relationship.

The present findings will promote an understanding of typical and atypical infant development through the formation of parent-infant bonding based on physical interaction. An important future study would be to conduct the hold/hug task in infants at high familial risk for autism spectrum disorder (ASD). Although ASD is rarely diagnosed this early, it has been reported that altered social communication with caregivers can be retrospectively seen as early as the later part of the first year (Elsabbagh and Johnson, 2010, Wan et al., 2019), when early signs of morphological brain development differences, such as the hyper-expansion of the cortical surface area, were observed in high-risk infants who were later diagnosed with autism (Hazlett et al., 2017). Thus, infants at high-risk for ASD who are older than four months old may exhibit a characteristic HRV reaction in the present hold/hug task with their parents or strangers.

Taken together, our results indicate that infants older than 4 months old may perceive hugs from their parents as a comfortable and relaxing experience, as opposed to holds by their parents and hugs from strangers. The parent-infant hug may make both the infants and parents feel relieved and happy, which leads to foster the parent-infant bonding.

### Limitations of the Study

One limitation of this study is that the hug/hold experiment accompanies large postural changes that occur in infants, which may affect infants' HRV. The baroreflex sensitivity of infants increases progressively over the first 6 months of age to reach the adult level (Yiallourou et al., 2010). An active baroreflex in response to being lifted out of the crib may disturb the RRI reaction during the hold/hug experiment.

We designed the hug/hold session protocol with the combination of 20-s tasks to minimize confounding factors and to enable all participants to do in the same way. However, a longer hug experiment may give us more detailed results such as the difference between mother-infant and father-infant pairs corresponding to the time spent in child care. In addition, a longer recording of electrocardiogram (ECG) of both infant and parent allows us to examine frequency domain and synchrony between infant and parent.

Another limitation of this study is that we cannot provide a mechanistic explanation of infant's responses specific to parental hugs. Compared with adults, children, especially first-year children, are difficult to be examined due to poor communication and technical limitations, which will be a challenge for future research.

## Methods

All methods can be found in the accompanying Transparent Methods supplemental file.

## Supplemental Information

Document S1. Transparent Methods, Figures S1–S6, and Tables S1–S3
