# Supplementary material for: Infants Show Physiological Responses Specific to Parental Hugs
Source: iScience. 2020 Apr 6;23(4):100996. doi: 10.1016/j.isci.2020.100996 (PMC7360522; doi:10.1016/j.isci.2020.100996)
Supplement: Document S1. Transparent Methods, Figures S1–S6, and Tables S1–S3 [file mmc1.pdf]

iScience, Volume *23*

Supplemental Information

Infants Show Physiological

Responses Specific to Parental Hugs

Sachine Yoshida, Yoshihiro Kawahara, Takuya Sasatani, Ken Kiyono, Yo Kobayashi, and Hiromasa Funato

Supplemental Figures

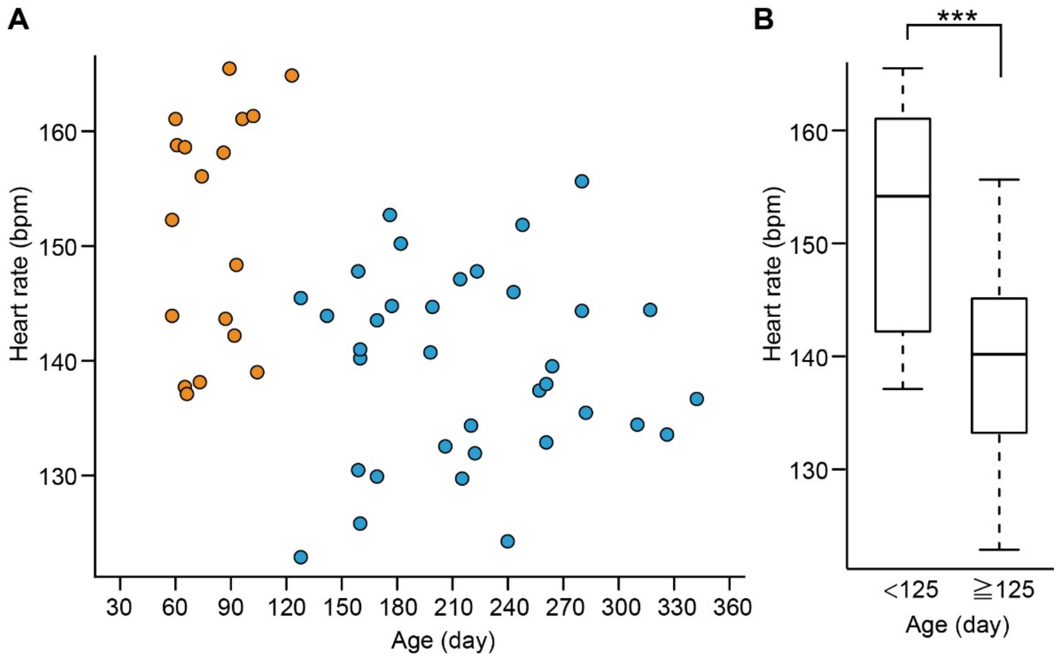

Figure S1. Heart rate in undisturbed infants in the crib. Related to Figure 1.

(A) The mean heart rate of infants were shown (orange circles: younger than 125 days old, blue circles: older than 125 days old). (B) The heart rate of infants older than 125 days old was lower than those younger than 125 days old. Welch's t test. The boxes represent the 25th, median, and 75th percentiles, and the whiskers represent the lowest or highest data within 1.5× interquartile range from the 25th or 75th percentile. The same data set with Figure 1 was analyzed. n = 53 (males = 28, females = 25). *** $p < 0.001$.

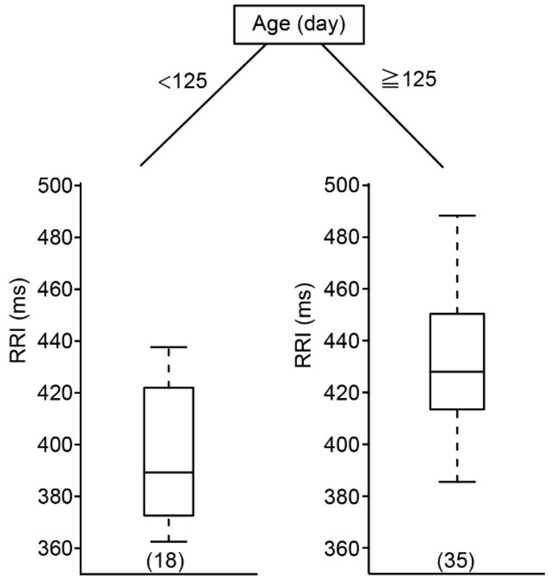

Figure S2. Decision tree analysis of infant RRI. Related to Figure 1.

CART analysis classifies mean RRI at 125 days old. Numbers in parentheses indicate the number of infants. Box plots indicate median (horizontal bar) values, 25th quartile and 75th quartile (box), and minimum and maximum (whiskers).

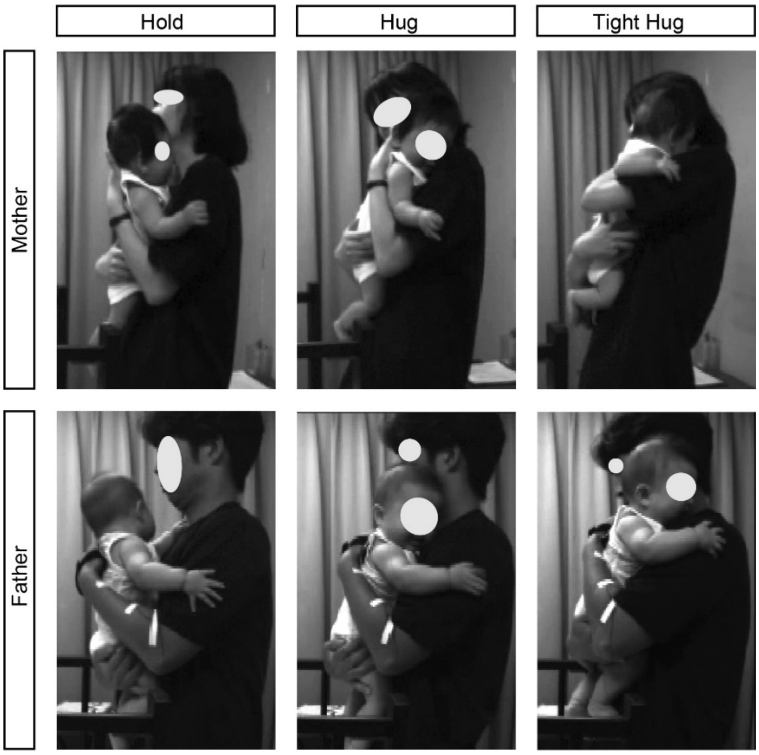

Figure S3. Representative postures of mother- and father-infant holding/hugging tasks. Related to Figure 2.

(Upper row) The mother holds, hugs, and hugs tightly her infant who is 104 days old. (Lower row) The father holds, hugs and hugs tightly his infant who is 177 days old.

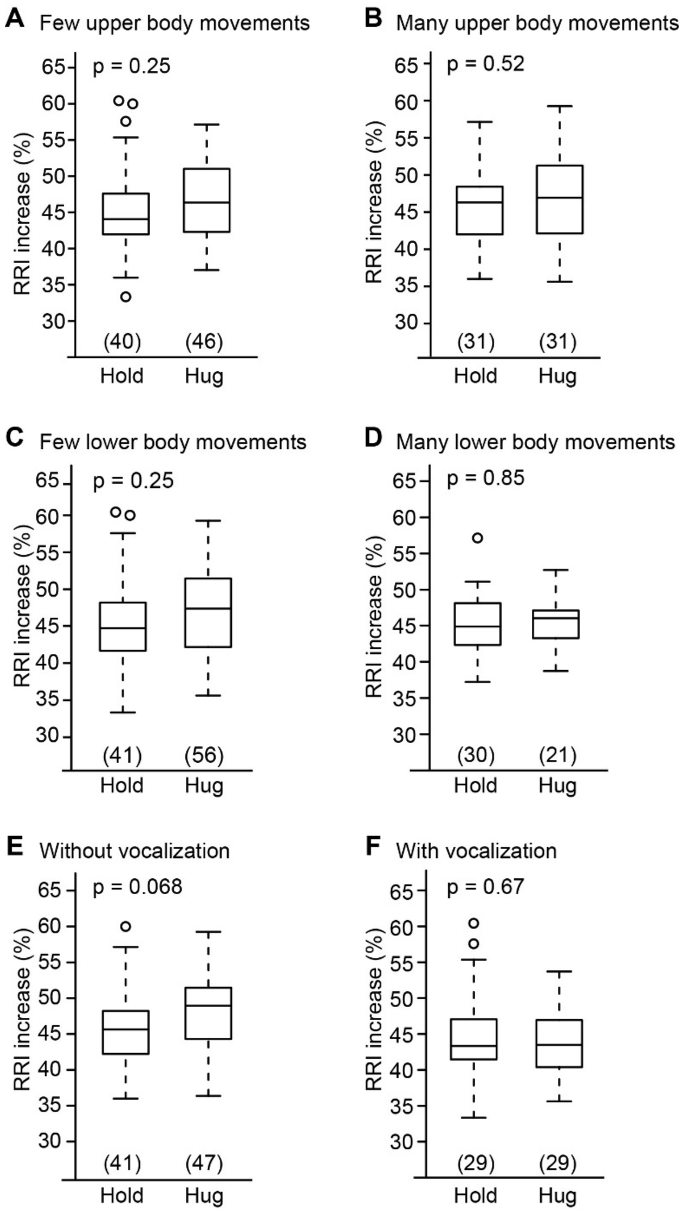

Figure S4. RRI increase ratio and infant activity during hold and hug. Related to Figure 3.

(A, B) RRI increase ratios during holding and during hugging were compared in infants who showed few (A) or many (B) upper body movements in the crib immediately before each task. (C, D) RRI increase ratios during holding and during hugging were compared in infants who showed few (C) or many (D) lower body movements in the crib immediately before each task. (E, F) RRI increase ratios during holding and during hugging were compared in infants who emitted vocalization (F) or not (E) in the crib immediately before each task. The boxes represent the 25th, median, and 75th percentiles, and the whiskers represent the lowest or highest data within 1.5× interquartile range from the 25th or 75th percentile. Numbers in parentheses indicate the number of infants.

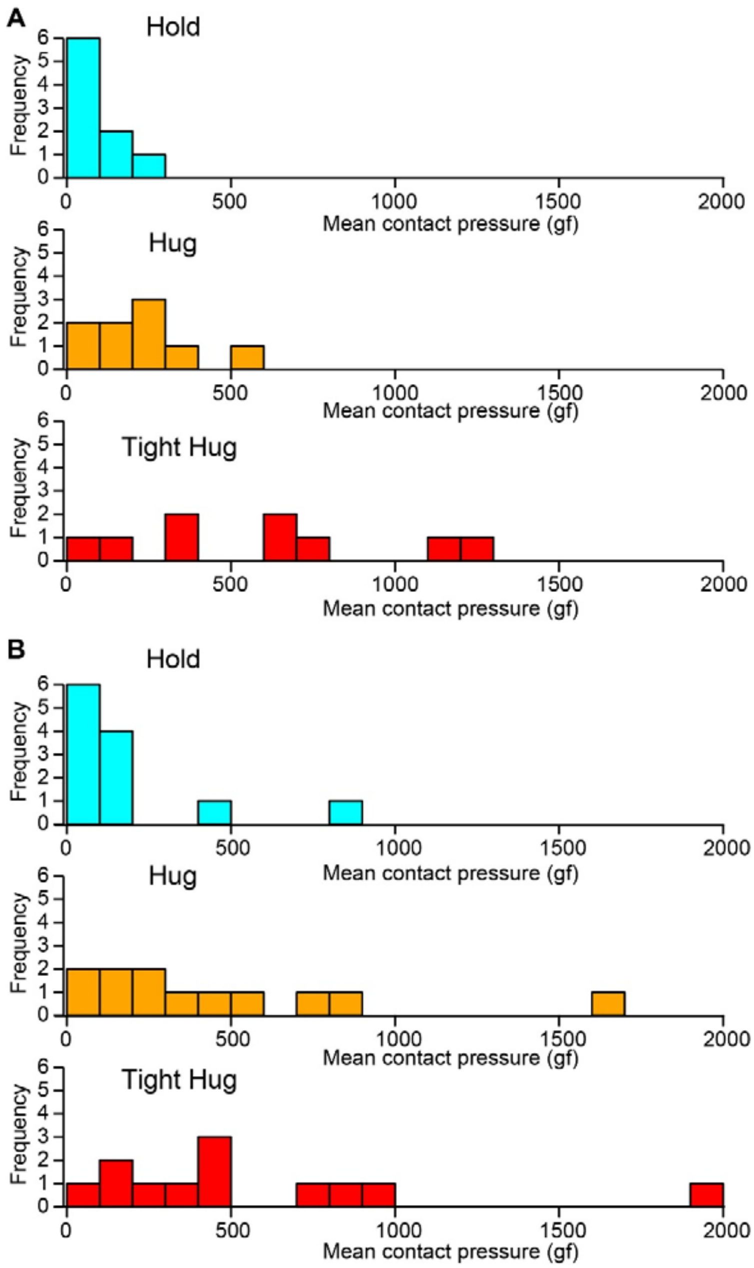

Figure S5. Contact pressure of the mother's hand on her infant's back during hold/hug. Related to Figure 6.

(A,B). Histograms show the mean contact pressure of the mother's hand on her infant under 125 days old (A) (n = 9 in each task) and over 125 days old (B) (n = 12 in each task) during a hold, hug and tight hug. Distribution patterns were compared using the Wilcoxon rank sum test with Holm's adjustment. (A) p = 0.0083 in Hold vs. Tight hug, p = 0.038 in Hold vs. Hug, p = 0.040 in Hug vs. Tight hug. (B) p = 0.0087 in Hold vs. Tight hug, p = 0.020 in Hold vs. Hug, p = 0.38 in Hug vs. Tight Hug.

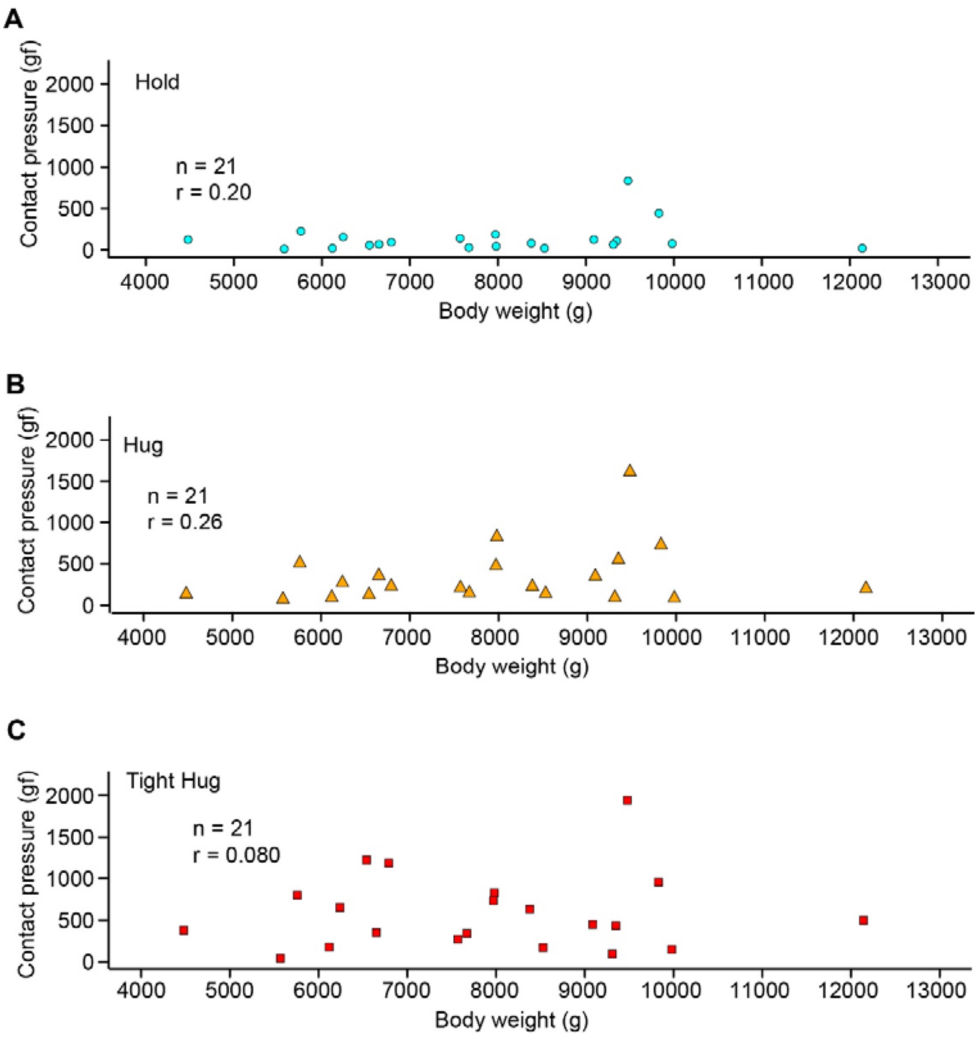

Figure S6. Contact pressure of the mother's hand and infant's body weight. Related to Figure 6.

(A-C) The mean contact pressure of the mother's hand on the infant's back during a hold (A), a hug (B), and a tight hug (C) were plotted against the infant's body weight.

Supplemental Tables

Age group	Variable	*B*	SE *B*	β (p-value)
< 125 days	Sex	-1.64	1.57	-0.12 (0.30)
	Body weight	-0.11	1.16	-0.010 (0.93)
	1st child or not	1.20	1.20	0.11 (0.32)
	Previous task type	-1.77	1.28	-0.15 (0.17)
	Task type	-2.70	0.95	-0.30 (0.0057) **
≥ 125 days	Sex	1.40	0.82	0.12 (0.090)
	Body weight	-0.14	0.40	0.024 (0.72)
	1st child or not	0.72	0.85	0.058 (0.40)
	Previous task type	0.20	0.85	0.016 (0.82)
	Task type	-1.55	0.50	-0.21 (0.0023) **

Table S1. Summary of multiple regression analysis for variables predicting RRI increase ratio. Related to Figure 3.

The unstandardized beta (*B*), the standard error for the unstandardized beta (SE *B*), and the standardized beta (β). **p < 0.01.

Variable	*B*	SE *B*	β (p-value)
Sex	0.30	1.16	0.027 (0.79)
Body weight	-0.22	0.62	-0.039 (0.72)
A hug was the 1st task or not	0.13	1.28	0.011 (0.92)
1st child or not	1.14	1.27	0.095 (0.37)
Head movement type	-6.47	1.32	-0.51 (< 0.0001) ****

Table S2. Summary of multiple regression analysis for variables predicting RRI increase ratio during hugging. Related to Figure 3.

The unstandardized beta (*B*), the standard error for the unstandardized beta (SE *B*), and the standardized beta (β). ****p < 0.0001.

Working time (h) (mean ± SD)	Childcare frequency (mean ± SD)
12.29 ± 2.22	2.14 ± 1.07

Table S3. Information about father participants. Related to Figure 7.

Based on our questionnaire survey, fathers' participation in childcare was assessed in a 4-point scale. 1, Taking care of infants only on days off; 2, Taking care of infants for 1-2 days on working days; 3, Taking care of infants for 3-5 days on working days; 4, Taking care of infants every day.

Transparent Methods

Participants

Mothers and fathers were recruited through advertisements during regular events held for postpartum parents at Toho University Omori Medical Center and local childcare support facilities. None of the participants suffered from serious physical and mental diseases. One infant had a Japanese mother and a British father, but all other infants had Japanese parents. All female participants were full-time homemakers or were on maternity leave. The body weight at the birth of the infants was 3.12 ± 0.33 kg. The age of the mothers, fathers, and female strangers were 33.2 ± 4.7, 35.8 ± 4.4, and 33.1 ± 4.0 years old, respectively. All participants that served as female strangers had childbirth experience. In mother/female stranger experiments, the difference in infants' ages was 11.5 ± 1.5 days. All father participants were Japanese and lived with their infants and worked outside the home, spending less time with their infants than mothers. The fathers' working hours and childcare frequency are summarized in Table S3. All experiments were approved by the ethical committee of the Faculty of Medicine at Toho University (A19055_A18121_A18036_A17065_A16095_27010_26110_26091).

Infants for RRI measurements

We excluded infants who cried before or during undisturbed conditions, holding, hugging or tight hugs. In the undisturbed condition, 53 infants (males (m) = 28, females (f) = 25) aged 58 to 342 days old were subjected to HRV analyses. In the mother-infant study, 136 infants aged 39 to 365 days old participated in the experiment (m = 78, f = 58). After exclusion, 110 (m = 62, f = 48), 109 (m = 61, f = 48) and 80 (m = 47, f = 33) infants were analyzed for holding, hugging, and tight hugging experiments, respectively. In the father-infant study, 13 (m 6, f 7), 14 (m 7, f 7), and 12 (m 6, f 6) infants more than 125 days old were analyzed for holding, hugging and tight hugging experiments, respectively. In the stranger-infant study, the number of infants over 125 days old analyzed in holding, hugging and tight hugging experiments were 12 (m = 10, f = 2), 13 (m = 9, f = 4) and 12 (m = 9, f = 3), respectively.

Pressure measurement of the mother's hand on the infant

Of the infants who were subjected to RRI measurements in the mother-infant study, 62 infants (m = 37, f = 25) participated in the pressure measurement. We excluded the data from crying infants and those including measurement failure. We analyzed 21 (m = 13, f = 8) infants aged 55 to 300 days old.

A flexible pressure sensor (FSR406, Interlink Electronics Inc.) was attached to a soft cloth and connected to a microcomputer (Arduino UNO) using conductive threads and processed wires. The microcomputer was connected to a PC via a USB 2.0 cable. Calibration at 0, 500 and 1000 g was performed before every recording. The sensor was attached to the mother's palm of the arm that is usually used to support the infant's back when the mother holds her infant (Figures 2D and 6A). The pressure was recorded at a sampling rate of 100 Hz. We described the load applied to the pressure sensor as contact pressure (gf). The pressure sensor was pressed three times at the beginning of and at the end of recording to create an indicator to be able to synchronize with video and ECG data.

ECG and acceleration sensor recording

The studies were performed between 10 AM and 3 PM. The rectangular room (263 cm x 377 cm) for the holding/hugging experiment had plain grayish walls on two sides and plain grayish curtains on two sides. A crib was placed in the room, 57 cm away from the walls, that had a mattress (120 cm x 69 cm) covered with a white bed sheet and slatted sides (36 cm high). To improve visibility, part of each slatted side was cut off and replaced with a transparent acrylic plate (64 cm x 68 cm). The room temperature was maintained at approximately 25°C. The bedsheet was changed between every infant, and the clothes were washed before each use.

Three disposable ECG electrode patches (Nihon Kohden, Japan) were placed on the chest of the mothers and fathers. The infants were changed to plain baby clothes, and three disposable ECG electrode patches (Nihon Kohden, Japan) and one acceleration sensor (Nihon Kohden, Japan) were placed on the upper chest with sticky tape (Figure 2A). ECG and acceleration sensors continuously measured signals through the experiment. All experiments were videotaped from the side and from above using Handycam camcorders (Sony, Japan) and BIMUTAS-Video software (Kissei Comtec, Japan). Acceleration data were obtained using BIMUTAS-Video software (Kissei Comtec, Japan). Since the duration of each holding/hugging task is not exactly 20 seconds, the evaluators determined the start and the end of the holding/hugging task for the heart rate variable based on video images.

Holding/hugging experimental procedure

When mothers and fathers arrived at the university laboratory, the experimental procedure was explained to parents, and they signed an informed consent form. We also obtained consent to use privacy-protected photos and videos by hiding participants' faces. The parents did not eat, drink or feed their infant 30 minutes before the experiment. The parents did not use any fragrance and a wristwatch. Before the experiment, the mothers and fathers changed clothes, into a plain shirt with short sleeves.

First, each infant was allowed to move freely in the crib with all the slatted sides up and measured for ECG for 1 min to establish an undisturbed condition in which the parent did not talk to or look into the eyes of their infants. Then, the parent lowered one side of the crib and stood still close to the crib for 20 seconds. Next, the parent was randomly instructed to either hold or hug their infant for 20 seconds. We decided the holding and hugging times based on preliminary hold/hug trials revealing that infants tended to become fussy around 30 seconds. The preliminary trials also revealed that the parents tended to show unnatural holding/hugging postures by the brief instructions without any examples such as hold, hug, and hug tightly. To allow the parents to hold/hug their infant as usual, the experimenter gave a demo of holding/hugging tasks using a cushion likening an infant with three detail instructions before the measurements: 1) hold the infant as usual, 2) hug the infant while thinking that the infant is adorable, not mechanically, and 3) hold the infant very tightly as the parent could run fast while holding the infant. In the present holding/hugging tasks, several common features were observed through each parent-infant pair (Figures 2D and S3). When the parent held the infant vertically, with one arm on the bottom and the other arm on the back, the infant was able to easily move the head and the upper body. There were often more space between infants' heads and parents' upper body parts during holding than during hugging and tight hugs. Since the parents were in close contact with the infant's face and upper body during hugging and hugging tightly, the infant tended to be unable to move their face and body. The most part of parents' and infants' faces were overlapped during tight

hugs compared to during holding and hugging. The parents did not rock the infant or talk to them during the experiment. When each 20-second holding/hugging task ended, the infant was placed back in the crib and was allowed to move freely for 20 seconds before the next task began. When the infant was placed back in the crib, the parents did not touch, talk to and look into the eyes of their infants. To examine responses to a female stranger, two pairs of similarly aged infants and their mothers came into the lab. One mother performed the same procedure with the other mother's infant. In some infants, the respiration rate was measured by visual confirmation during a 20-sec hug and hold because of the technical difficulty of respiration sensor attachment around the infant's chest.

Two of three observers (SY, RM, and MT) described infants' behaviors, including vocalizations and movements of the head, upper body, and lower body, during each task. Using a stopwatch, the observers measured the movement times of each body part and then classified these infants into "few" and "many" movements groups depending on whether the infant moved the body part for more than half of the task time (approximately 10 seconds) or less. Observation and classification were conducted independently by two observers. Interrater reliability was calculated on 25 % of the data using Cohen k and was statistically acceptable (k = 0.90 in head movement, k = 0.92 in upper body movement, k = 0.94 in lower body movement, and k = 0.97 in vocalization).

ECG recording and analysis

ECG signals were recorded at a sampling rate of 1000 Hz (Nihon Kohden). We calculated time domain parameters such as mean RRI and RMSSD. Because successive RRI differences hardly exceed 50 ms in awake first-year infants, we did not use pNN50, one of the major time domain parameters. Previous studies in preterm neonates and toddlers set the shorter threshold as pNN5 (Reulecke et al., 2012) and pNN10 (Billeci et al., 2018), respectively. Different from pNN-type parameter in which both RRI increases and decreases were counted, we defined the RRI increase ratio as a percentage of successive RRIs that are longer than the previous RRI.

As frequency domain parameters, we used LF and HF calculated by fast Fourier transformation. LF- and HF-power bands are the absolute values of the low-frequency band (0.04 to 0.15 Hz) and the high-frequency band (0.15 Hz to 0.4 Hz) in adults (Task Force of The European Society of Cardiology and the North American Society of Pacing and Electrophysiology, 1996). We used 0.02-0.2 Hz as the LF range and 0.2-2 Hz as the HF range, considering the much higher heart rates in infants, as previously described (Chatow et al., 1995; Longin et al., 2005; Patzak et al., 1996; Rosenstock et al., 1999). The HF band reflects parasympathetic activity and corresponds to the heart rate variations related to the respiratory cycle (Shaffer et al., 2017). The ratio of LF to HF power (LF/HF ratio) may estimate the sympathetic dominance over parasympathetic factors (Shaffer et al., 2017). Heart rate variables and related parameters were calculated using R software.

We quantified the RRI increase ratio during holding, hugging, and tight hugging tasks. The RRI increase ratio is the percentage of cardiac cycles that have a longer RRI than the previous cycle during the 20 second-hug/hold task. Decision tree classification using binarized RRI data was performed to examine whether the head movement can predict RRI changes. The RRI increase ratio was binarized depending on whether it was over or under the reference value. The reference value (44.22 %) was the mean of the median RRI increase ratio for infants under 125 days old (42.15 %) and infants over 125 days old (46.29 %).

Statistical analysis

The data were preprocessed and visualized with Microsoft Excel 2016, R version 3.6.1 and Python 3.6. All statistical analyses were conducted using R. We performed CART analysis, Fisher's exact test, linear regression analysis, multiple regression analysis, multivariate analysis of variance (MANOVA) followed by one-way ANOVA and the Tukey-Kramer post-hoc test, Pearson's correlation test, Welch's t-test, Welch's ANOVA or Wilcoxon test. Significance was set at $p < 0.05$ after p-value correction by Holm's method.

Supplemental References.

Billeci, L., Tonacci, A., Narzisi, A., Manigrasso, Z., Varanini, M., Fulceri, F., Lattarulo, C., Calderoni, S., and Muratori, F. (2018). Heart Rate Variability During a Joint Attention Task in Toddlers With Autism Spectrum Disorders. Front. Physiol. *9*, 467.

Chatow, U.D.I., Davidson, S., Reichman, B.L., and Akselrod, S. (1995). Development and maturation of the autonomic nervous system in premature and full-term infants using spectral analysis of heart rate fluctuations. Pediatr. Res. *37*, 294–302.

Longin, E., Schaible, T., Lenz, T., and König, S. (2005). Short term heart rate variability in healthy neonates: Normative data and physiological observations. Early Hum. Dev. *81*, 663–671.

Patzak, A., Lipke, K., Orlow, W., Mrowka, R., Stauss, H., Windt, E., Persson, P.B., and Schubert, E. (1996). Development of heart rate power spectra reveals neonatal peculiarities of cardiorespiratory control. Am. J. Physiol. *271*, R1025-32.

Reulecke, S., Schulz, S., and Voss, A. (2012). Autonomic Regulation during Quiet and Active Sleep States in Very Preterm Neonates. Front. Physiol. *3*, 61.

Rosenstock, E.G., Cassuto, Y., and Zmora, E. (1999). Heart rate variability in the neonate and infant: Analytical methods, physiological and clinical observations. Acta Paediatr. Int. J. Paediatr. *88*, 477–482.

Shaffer, F., Ginsberg, J.P., and Shaffer, F. (2017). An Overview of Heart Rate Variability Metrics and Norms. Front. Public Heal. *5*, 258.

Task Force of The European Society of Cardiology and the North American Society of Pacing and Electrophysiology, .a (1996). Heart rate variability. Standards of measurement, physiological interpretation, and clinical use. Task Force of the European Society of Cardiology and the North American Society of Pacing and Electrophysiology. Eur. Heart J. *17*, 354–381.
